# A genotype imputation method for de-identified haplotype reference information by using recurrent neural network

**DOI:** 10.1371/journal.pcbi.1008207

**Published:** 2020-10-01

**Authors:** Kaname Kojima, Shu Tadaka, Fumiki Katsuoka, Gen Tamiya, Masayuki Yamamoto, Kengo Kinoshita

**Affiliations:** 1 Tohoku Medical Megabank Organization, Tohoku University, Sendai, Miyagi, Japan; 2 RIKEN Center for Advanced Intelligence Project, Chuo-ku, Tokyo, Japan; 3 School of Medicine, Tohoku University, Sendai, Miyagi, Japan; 4 Advanced Research Center for Innovations in Next-Generation Medicine, Tohoku University, Sendai, Miyagi, Japan; 5 Graduate School of Information Sciences, Tohoku University, Sendai, Miyagi, Japan; 6 Institute of Development, Aging and Cancer, Tohoku University, Sendai, Miyagi, Japan; La Jolla Institute for Allergy and Immunology, UNITED STATES

## Abstract

Genotype imputation estimates the genotypes of unobserved variants using the genotype data of other observed variants based on a collection of haplotypes for thousands of individuals, which is known as a haplotype reference panel. In general, more accurate imputation results were obtained using a larger size of haplotype reference panel. Most of the existing genotype imputation methods explicitly require the haplotype reference panel in precise form, but the accessibility of haplotype data is often limited, due to the requirement of agreements from the donors. Since de-identified information such as summary statistics or model parameters can be used publicly, imputation methods using de-identified haplotype reference information might be useful to enhance the quality of imputation results under the condition where the access of the haplotype data is limited. In this study, we proposed a novel imputation method that handles the reference panel as its model parameters by using bidirectional recurrent neural network (RNN). The model parameters are presented in the form of de-identified information from which the restoration of the genotype data at the individual-level is almost impossible. We demonstrated that the proposed method provides comparable imputation accuracy when compared with the existing imputation methods using haplotype datasets from the 1000 Genomes Project (1KGP) and the Haplotype Reference Consortium. We also considered a scenario where a subset of haplotypes is made available only in de-identified form for the haplotype reference panel. In the evaluation using the 1KGP dataset under the scenario, the imputation accuracy of the proposed method is much higher than that of the existing imputation methods. We therefore conclude that our RNN-based method is quite promising to further promote the data-sharing of sensitive genome data under the recent movement for the protection of individuals’ privacy.

## Author summary

Genotype imputation estimates the genotypes of unobserved variants using the genotype data of other observed variants based on a collection of genome data of a large number of individuals called a reference panel. In general, more accurate imputation results are obtained using a larger size of the reference panel. Although most of the existing imputation methods use the reference panel in an explicit form, the accessibility of genome data is often limited due to the requirement of agreements from the donors. We thus proposed a new imputation method that handles the reference panel as its model parameters by using bidirectional recurrent neural network. Since it is almost impossible to restore genome data at the individual-level from the model parameters, they can be shared publicly as the de-identified information even when the accessibility of the original reference panel is limited. We demonstrate that the proposed method provides comparable imputation accuracy with the existing methods. We also considered a scenario where a part of the genome data is made available only in de-identified form for the reference panel and have shown that the imputation accuracy of the proposed method is much higher than that of the existing methods under the scenario.

This is a *PLOS Computational Biology* Methods paper.

## Introduction

The development of high-throughput sequencing technologies enabled the construction of genotype data with base-level resolution for more than one thousand individuals. The collection of haplotypes from such large-scale and high-resolution genotype data is known as a haplotype reference panel, and one of the major applications of the haplotype reference panels is genotype imputation. SNP array technology can acquire the genotype data at a much lower cost than that required for sequencing, and hence SNP array is considered to be suitable for studies requiring genotype data of a significantly higher number of individuals, such as genome-wide association studies (GWAS), trait heritability analysis, and polygenic risk score estimation. Although genotype data obtained using the SNP array is limited to the designed markers, genotype data with sequencing-level resolution obtained from genotype imputation enables the detection of more trait-related variants in GWAS and more accurate estimation of trait heritability and polygenic risk scores [1–3]. The current imputation methods such as Impute2 [4], Minimac3 [5], and Beagle5.1 [6] are based on a model introduced by Li and Stephens in which a new haplotype can be represented by applying mutations and recombinations to the haplotypes present in the haplotype reference panel [7]. The imputation methods based on the Li and Stephens model consider phased genotypes obtained using SNP array or other genotyping technologies as input genotype data, and estimate the haplotypes that match with the input genotype data by considering the recombinations of haplotypes present in the haplotype reference panel. Genotypes of unobserved variants are then obtained from the estimated haplotypes.

Although the imputation methods based on the Li and Stephens model require a haplotype reference panel as in an explicit form, the accessibility of haplotype data is often limited, due to the requirement of agreements from the donors for public use. For example, the Northeast Asian Reference Database (NARD) has a haplotype reference panel comprised of 1,779 northeast Asian individuals, but the haplotype panel is not publicly available, and thus can be used only in the NARD imputation server [8]. Thus, in order to use publicly unavailable haplotypes for more accurate imputation, we must send the input genotype data to other research institutes having their own closed haplotype data. However, the input genotype data itself also often has some limitations for external use due to the informed consent policy. One solution for this issue is to handle the haplotype information in de-identified form such as summary statistics or model parameters from which the restoration of genotype data at an individual-level is almost impossible. E.g., the aggregation of summary statistics has been considered in GWAS for the calculation of p-values without sharing individual-level genotypes among cohorts [9]. There also exist correction methods for sample overlap in GWAS without sharing individual-level genotypes [10–12]. Already, there exists a supervised learning-based imputation method that imputes genotypes using support vector machine (SVM) trained with the haplotype reference panel [13]. Although the SVM-based method requires less computational time and less memory space, its imputation accuracy is not sufficient when compared to that of the imputation methods based on the Li and Stephens model. Recent development of deep learning technologies including recurrent neural networks (RNN) provides significant improvements in various fields such as image classification [14], image detection [15], natural language understanding [16], and speech and video data recognition [17]; the deep learning technologies are therefore more promising for devising imputation methods that can handle the haplotype information as the model parameters.

In this study, we proposed a new imputation method based on bidirectional recurrent neural network that takes the phased genotypes as input data and returns probabilities of alleles for the unobserved variants. In the proposed method, the information of the haplotype reference panel is parameterized as model parameters through the training step, and the haplotype data itself is not explicitly used for the imputation. We have considered binary vectors that represent allele patterns of observed variants in the haplotype reference panel, for the input feature vectors of the bidirectional RNN. We have applied the dimensionality reduction to the binary vectors using the kernel principal component analysis in order to reduce the length of the feature vectors and avoid the restoration of the genotype data. For RNN cells, we have considered long short-term memory (LSTM) [18] and gated recurrent unit (GRU) [19] in the proposed method. We also proposed a hybrid model obtained by combining two bidirectional RNN models trained for different minor allele frequency (MAF) ranges as well as a new data augmentation process for more robust and accurate estimation. Since it is difficult to restore the genotype information at an individual-level from the model parameters, the model parameters can be shared for public use even if the haplotype data used for training is not permitted for public use.

In the performance evaluation based on the comparison with existing imputation methods, we have used haplotype datasets from the 1000 Genomes Project (1KGP) [20] and the Haplotype Reference Consortium (HRC) [21]. Overall, the imputation accuracy of the proposed model in *R*^2^ is comparable with that of existing imputation methods based on the Li and Stephens model in both of the datasets. For low frequency variant sites, the imputation accuracy of the proposed model in *R*^2^ tends to be lower than that of the imputation methods in both of the datasets. We also have considered a scenario where some haplotypes are made available only in de-identified form for the haplotype reference panel due to the issue of limited accessibility. Under this scenario, some haplotypes are not made available for the existing imputation methods based on the Li and Stephens model, while the proposed method and the above-mentioned SVM-based imputation method can use all the haplotypes. For the evaluation under the scenario, where the haplotypes are obtained from the 1KGP dataset, the imputation accuracy of the proposed method is higher than that of the existing imputation methods based on the Li and Stephens model as well as the SVM-based imputation method, at least for variants with MAF ≥ 0.005. In addition, the imputation accuracy of the proposed method is higher than that of the SVM-based imputation method in the entire MAF range for all the experiments.

## Methods

**Fig 1:**
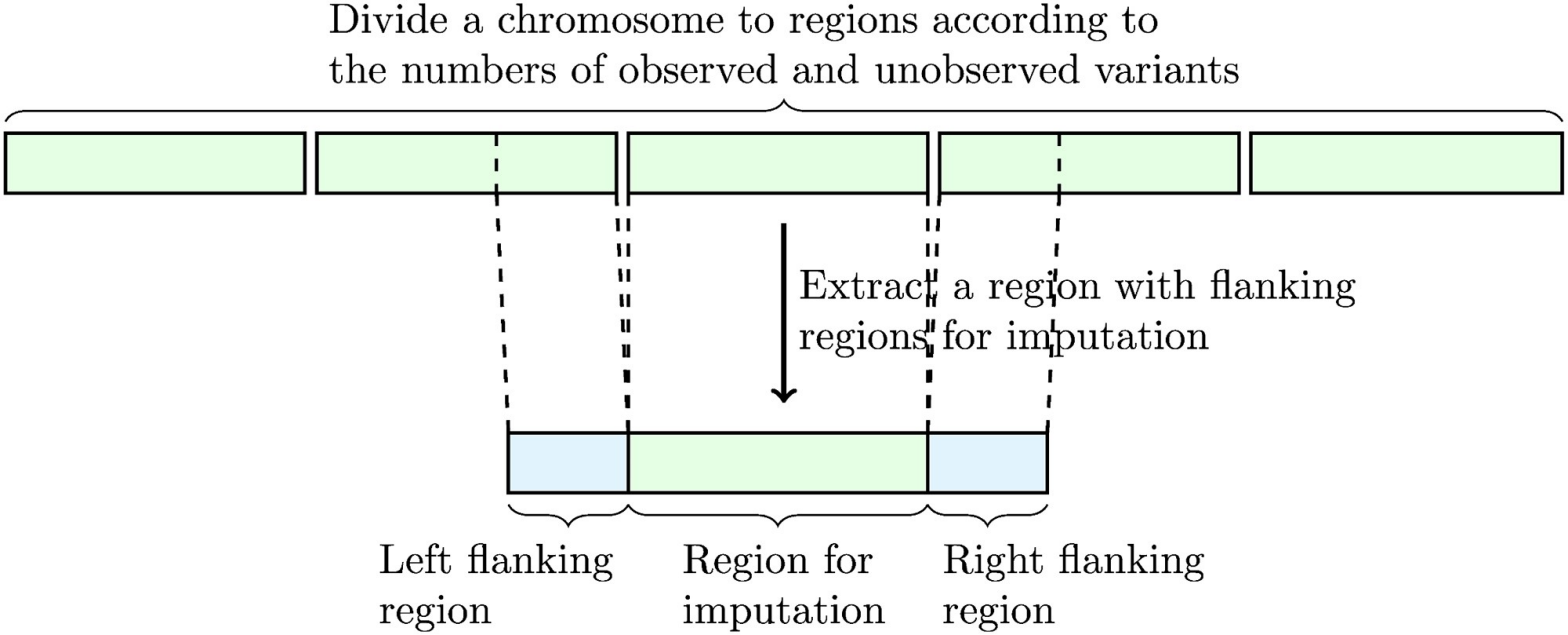
An illustration of division of a chromosome to regions according to the numbers of observed and unobserved variants for imputation.

Let *v*_*i*_ and *u*_*j*_ be the *i*th observed variant and *j*th unobserved variant, respectively. We assume that the orders of the observed and unobserved variants are respectively sorted with their genomic positions, and hence *p*(*v*_*i*_) ≤ *p*(*v*_*j*_) and *p*(*u*_*i*_) ≤ *p*(*u*_*j*_) are satisfied for *i* < *j*, where *p*(⋅) is a function that returns the position of the input variant. We use RNN models to capture the transition of haplotype information on the sorted observed variants to estimate alleles for unobserved variants. We divide a chromosome to regions according to the numbers of observed and unobserved variants as shown in Fig 1 due to the restriction of memory usage for the RNN model. We limit the maximum numbers of observed and unobserved variants in each region to 100 and 1,000 in our experiment, respectively, and each region is extended up to the limit in the division process. Each divided region has flanking regions in the upstream and downstream directions, and only the observed variants are considered in the flanking regions. The proposed method takes the haplotype comprised of observed variants and imputes unobserved variants for each region, and imputation results from the divided regions are concatenated for the final imputation result. In the following subsections, we describe the model structure of the proposed method for each region, the extraction of input feature vectors for the model, and details of the procedures for training the model.

### Model structure

**Fig 2:**
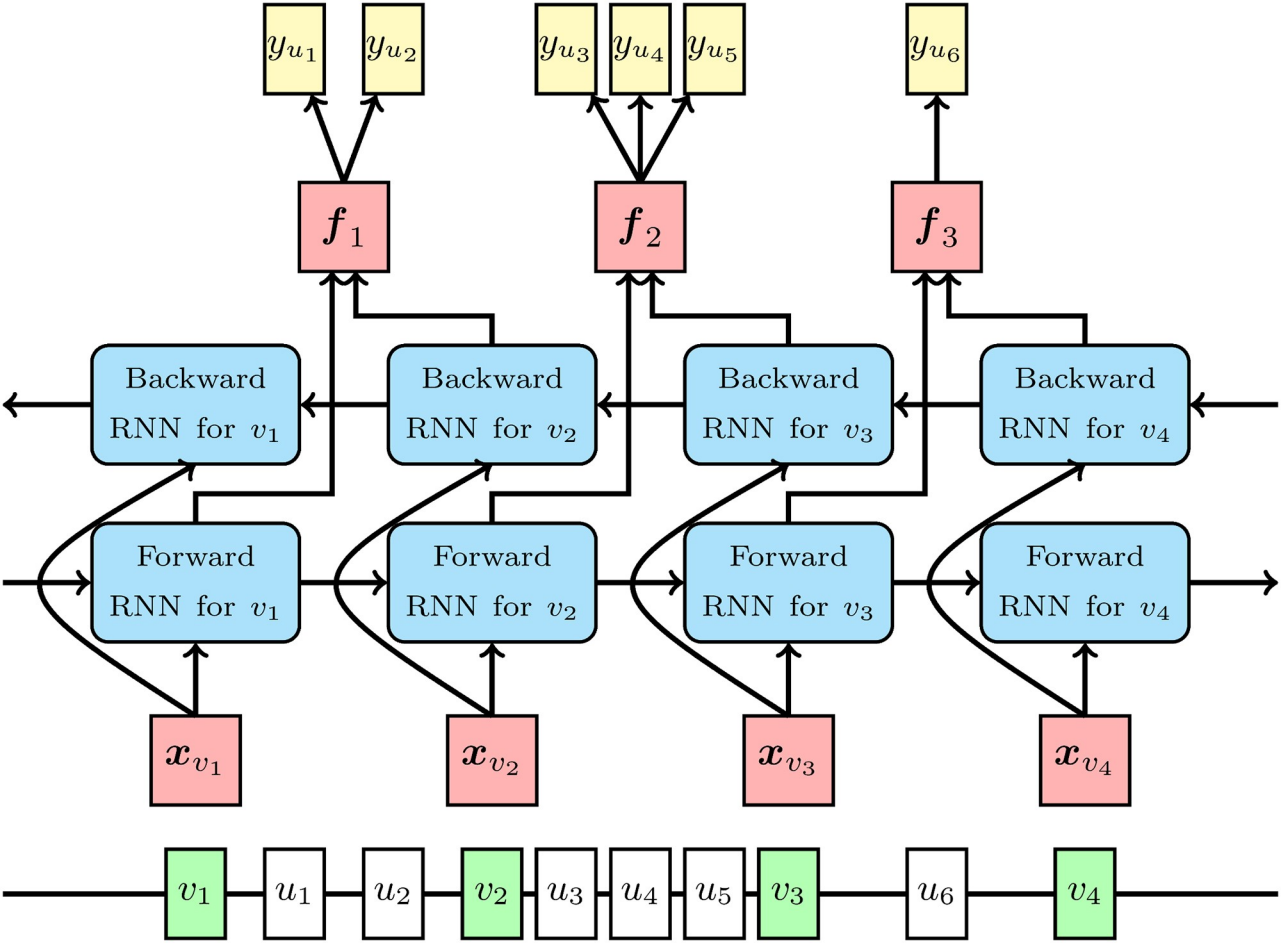
The overall model structure of the proposed method. The line in the bottom of the figure indicates a genome sequence where observed variants are in green square and unobserved variants are in white square. Forward and backward RNNs are built on the observed variants. $x_{v_{i}}$ is the input feature vector of the forward and backward RNNs for observed variant *v*_*i*_. ***f***_*i*_ is the vector from the concatenation of the output of the forward RNN for observed variant *v*_*i*_ and the output of the backward RNN for observed variant *v*_*i*+1_. $y_{u_{i}}$ is a binary variable indicating the allele for unobserved variant *u*_*i*_.

**Fig 3:**
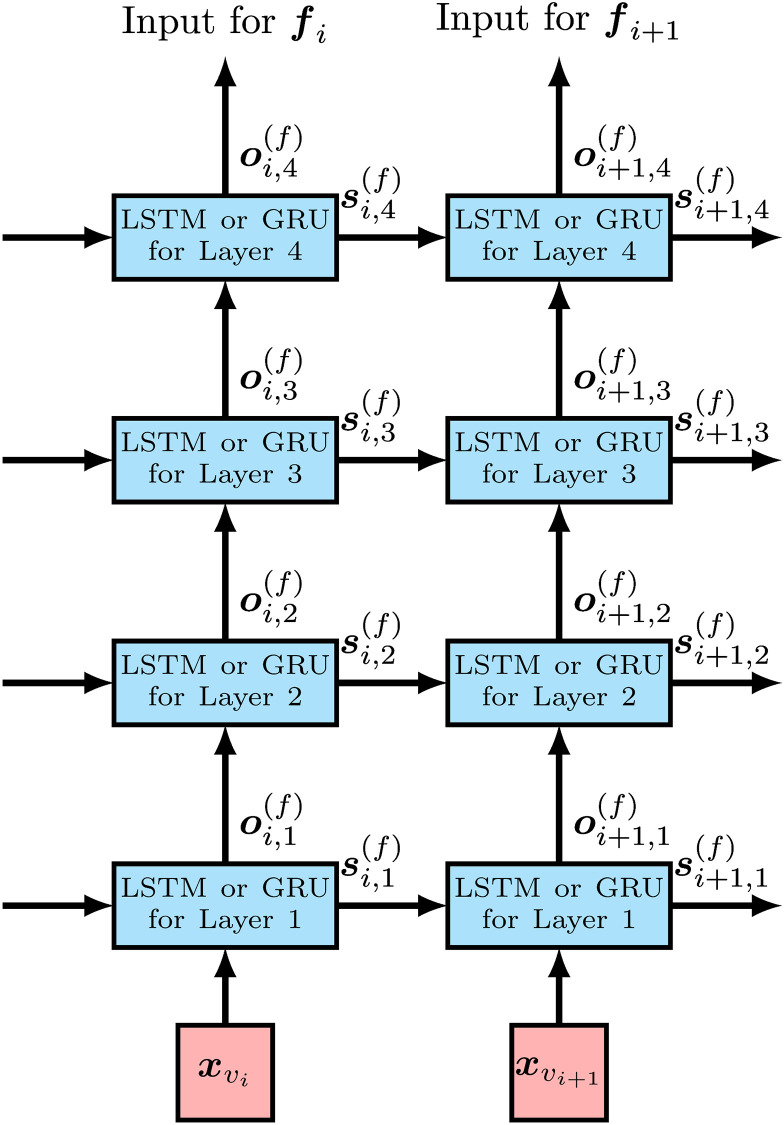
The structure of the forward RNN for each observed variant for the case of four stacked RNN cells. $x_{v_{i}}$ and $x_{v_{i+1}}$ are input feature vectors for observed variants *v*_*i*_ and *v*_*i*+1_, respectively. $s_{i,j}^{\left(f\right)}$ is the state of the RNN cell of the *j*th layer for observed variant *v*_*i*_ and used as the input of the state for the RNN cell of the *j*th layer for observed variant *v*_*i*+1_. The output of the RNN cell of the top layer, ***o***_*i*,4_, is handled as the output of RNN for each observed variant.

We assume that both observed and unobserved variants are biallelic; i.e., their alleles are represented by one and zero. Let *m* be the number of the observed variants in a divided region. We also let *m*_*l*_ and *m*_*r*_ respectively be indices of the left most and right most observed variants in the region without the left and right flanking regions. We build a bidirectional RNN on the observed variants for each divided region as shown in Fig 2. A forward RNN is built on observed variants $v_{1},\ldots ,v_{m_{r}}$, and observed variants $v_{m_{r}+1},\ldots ,v_{m}$ in the right flanking region are not included, since the variants in the right flanking region are not required for imputing the unobserved variants in the forward direction. A backward RNN is built on observed variants $v_{m_{l}},\ldots ,v_{m}$, and similarly to the forward RNN, $v_{1},\ldots ,v_{m_{l}-1}$ in the left flanking region are not included. RNN cells for each observed variant of the forward and backward RNNs are stacked in the proposed model as shown in Fig 3, and LSTM and GRU are considered for RNN cells. $s_{i,l}^{\left(f\right)}$ and $o_{i,l}^{\left(f\right)}$ in Fig 3 are the state and output vectors of the RNN cell for the *l*th layer on observed variant *v*_*i*_ in the forward RNN, respectively. The length of $s_{i,l}^{\left(f\right)}$ is the same as the length of $o_{i,l}^{\left(f\right)}$ for *l* ≥ 1. We call the length the number of hidden units, and denote it by *H*. $s_{i,l}^{\left(f\right)}$ and $o_{i,l}^{\left(f\right)}$ are obtained recursively for *i* ∈ {1, …, *m*_*r*_} in the following manner:

$$\begin{array}{ccc} s_{i,l}^{\left(f\right)} & = & S_{l}^{\left(f\right)}\left(s_{i-1,l}^{\left(f\right)},o_{i,l-1}^{\left(f\right)}\right)\\ o_{i,l}^{\left(f\right)} & = & O_{l}^{\left(f\right)}\left(s_{i-1,l}^{\left(f\right)},o_{i,l-1}^{\left(f\right)}\right), \end{array}$$

where $S_{l}^{\left(f\right)}$ and $O_{l}^{\left(f\right)}$ are functions that represent the state and output vectors from the RNN cell of the *l*th layer, respectively. $S_{l}^{\left(f\right)}$ and $O_{l}^{\left(f\right)}$ are parameterized with learnable parameters of the RNN cell, and their actual forms are dependent on the type of the RNN cell such as LSTM and GRU. Note that the initial state $s_{0,l}^{\left(f\right)}$ is set to a zero vector of the length of *H*, and $o_{i,0}^{\left(f\right)}$ is set to the input feature vector for the *i*th observed variant $x_{v_{i}}$. We let *I* be the length of $x_{v_{i}}$, and hence the length of $o_{i,0}^{\left(f\right)}$ is also *I*. Details of the input feature vectors for the observed variants are described in the next subsection. For the backward RNN, we use the corresponding notations to those used in the forward RNN, and obtained

$$\begin{array}{ccc} s_{i,l}^{\left(b\right)} & = & S_{l}^{\left(b\right)}\left(s_{i+1,l}^{\left(b\right)},o_{i,l-1}^{\left(b\right)}\right)\\ o_{i,l}^{\left(b\right)} & = & O_{l}^{\left(b\right)}\left(s_{i+1,l}^{\left(b\right)},o_{i,l-1}^{\left(b\right)}\right), \end{array}$$

where *i* ∈ {*m*_*l*_, …, *m*}. Note that $s_{m+1,l}^{\left(b\right)}=0$ and $o_{i,0}^{\left(b\right)}=x_{v_{i}}$.

Let ***f***_*i*_ be a vector given by the concatenation of the output vectors of the forward and backward RNNs as shown in Fig 2:

$$\begin{array}{c} f_{i}=[\begin{array}{c} o_{i,L}^{\left(f\right)}\\ o_{i+1,L}^{\left(b\right)} \end{array}], \end{array}$$

where *L* is the number of layers in the model. Let $y_{u_{i}}$ be a binary value representing the allele of unobserved variant *u*_*i*_. The probability of $y_{u_{i}}=1$ is estimated by the following softmax function:

$$\begin{array}{c} \frac{\exp(a_{i,1}^{T}f_{\tilde{i}}+b_{i,1})}{\sum_{j=0}^{1}\exp(a_{i,j}^{T}f_{\tilde{i}}+b_{i,j})}, \end{array}$$

where ***a***_*i*,*j*_ and *b*_*i*,*j*_ are learnable parameters, and $\tilde{i}$ is the index that satisfies $p\left(v_{\tilde{i}}\right)\leq p\left(u_{i}\right)<p\left(v_{\tilde{i}+1}\right)$; i.e., $\tilde{i}$ is the index for the closest observed variant to *u*_*i*_ in the upstream region. For the case of *p*(*u*_*i*_) < *p*(*v*_1_), which occurs in the left most divided region, $f_{\tilde{i}}$ is given by $o_{1,L}^{\left(b\right)}$. Similarly, $f_{\tilde{i}}$ is given by $o_{m,L}^{\left(f\right)}$ for the case of *p*(*u*_*i*_) ≥ *p*(*v*_*m*_).

For the loss function for training the model parameters, we consider the sum of the weighted cross entropies over the unobserved variants as follows:

$$\begin{array}{c} \sum_{i=1}^{n}{\left(2{\text{MAF}}_{i}\right)}^{\gamma }(-y_{u_{i}}\frac{\exp(a_{i,1}^{T}f_{\tilde{i}}+b_{i,1})}{\sum_{j=0}^{1}\exp(a_{i,j}^{T}f_{\tilde{i}}+b_{i,j})}-\left(1-y_{u_{i}}\right)\frac{\exp(a_{i,0}^{T}f_{\tilde{i}}+b_{i,0})}{\sum_{j=0}^{1}\exp(a_{i,j}^{T}f_{\tilde{i}}+b_{i,j})}), \end{array}$$

where *n* is the number of the unobserved variants, *MAF*_*i*_ is the minor allele frequency of *u*_*i*_ in the training data, and *γ* is a hyperparameter to adjust the weights from MAF. The loss function with *γ* > 0 gives higher priority to higher MAF variants, while the loss function with *γ* < 0 gives higher priority to lower MAF variants.

#### Hybrid model comprised of models trained with different *γ*

Since the models trained on loss functions with *γ* > 0 and *γ* < 0 respectively give priority to higher and lower MAF variants, we consider a hybrid model obtained by the combination of the models trained with *γ* > 0 and *γ* < 0 for achieving higher accuracy in both high and low MAF variants. We hereafter call the model trained with *γ* > 0 “higher MAF model” and that with *γ* < 0 “lower MAF model”. For the hybrid model, we consider a 4-length vector ***g***_*i*_ for each unobserved variant *u*_*i*_ given by the combination of logits of these two models as follows:

$$\begin{array}{c} g_{i}=[\begin{array}{c} {\left(a_{i,1}^{\left(h\right)}\right)}^{T}f_{\tilde{i}}^{\left(h\right)}+b_{i,1}^{\left(h\right)}\\ {\left(a_{i,0}^{\left(h\right)}\right)}^{T}f_{\tilde{i}}^{\left(h\right)}+b_{i,0}^{\left(h\right)}\\ {\left(a_{i,1}^{\left(l\right)}\right)}^{T}f_{\tilde{i}}^{\left(l\right)}+b_{i,1}^{\left(l\right)}\\ {\left(a_{i,0}^{\left(l\right)}\right)}^{T}f_{\tilde{i}}^{\left(l\right)}+b_{i,0}^{\left(l\right)} \end{array}], \end{array}$$

where superscripts (*h*) and (*l*) indicate the variables and outputs of the higher and lower MAF models, respectively, We then estimate the probability of $y_{u_{i}}=1$ by the following softmax function for ***g***_*i*_:

$$\begin{array}{c} \frac{\exp(c_{i,1}^{T}g_{i}+d_{i,1})}{\sum_{j=0}^{1}\exp(c_{i,j}^{T}g_{i}+d_{i,j})}, \end{array}$$

where ***c***_*i*,*j*_ and *d*_*i*,*j*_ are learnable parameters. After the learning of the parameters of the higher and lower MAF models, we train ***c***_*i*,*j*_ and *d*_*i*,*j*_ in the loss function by the sum of the cross entropies as follows:

$$\begin{array}{c} \sum_{i=1}^{n}(-y_{u_{i}}\frac{\exp(c_{i,1}^{T}g_{i}+d_{i,1})}{\sum_{j=0}^{1}\exp(c_{i,j}^{T}g_{i}+d_{i,j})}-\left(1-y_{u_{i}}\right)\frac{\exp(c_{i,0}^{T}g_{i}+d_{i,0})}{\sum_{j=0}^{1}\exp(c_{i,j}^{T}g_{i}+d_{i,j})}). \end{array}$$

Note that the parameters of the higher and lower MAF models are fixed for training ***c***_*i*,*j*_ and *d*_*i*,*j*_.

#### Residual connections

For deep neural networks, gradients of parameters in backpropagation tend to vanish, and the sufficient training of the parameters fails due to the vanishing of gradients. In residual connections, only residues are calculated in each layer, and the vanishing gradient problem is avoided by skipping the main part of the data flow [22]. We thus consider RNN models with residual connections, which can be obtained by simple changes in the outputs of each layer for *l* > 1 as follows:

$$\begin{array}{ccc} o_{i,l}^{\left(f\right)} & = & O_{l}^{\left(f\right)}\left(s_{i+1,l}^{\left(f\right)},o_{i,l-1}^{\left(f\right)}\right)+o_{i,l-1}^{\left(f\right)},\\ o_{i,l}^{\left(b\right)} & = & O_{l}^{\left(b\right)}\left(s_{i+1,l}^{\left(b\right)},o_{i,l-1}^{\left(b\right)}\right)+o_{i,l-1}^{\left(b\right)}. \end{array}$$

**Fig 4:**
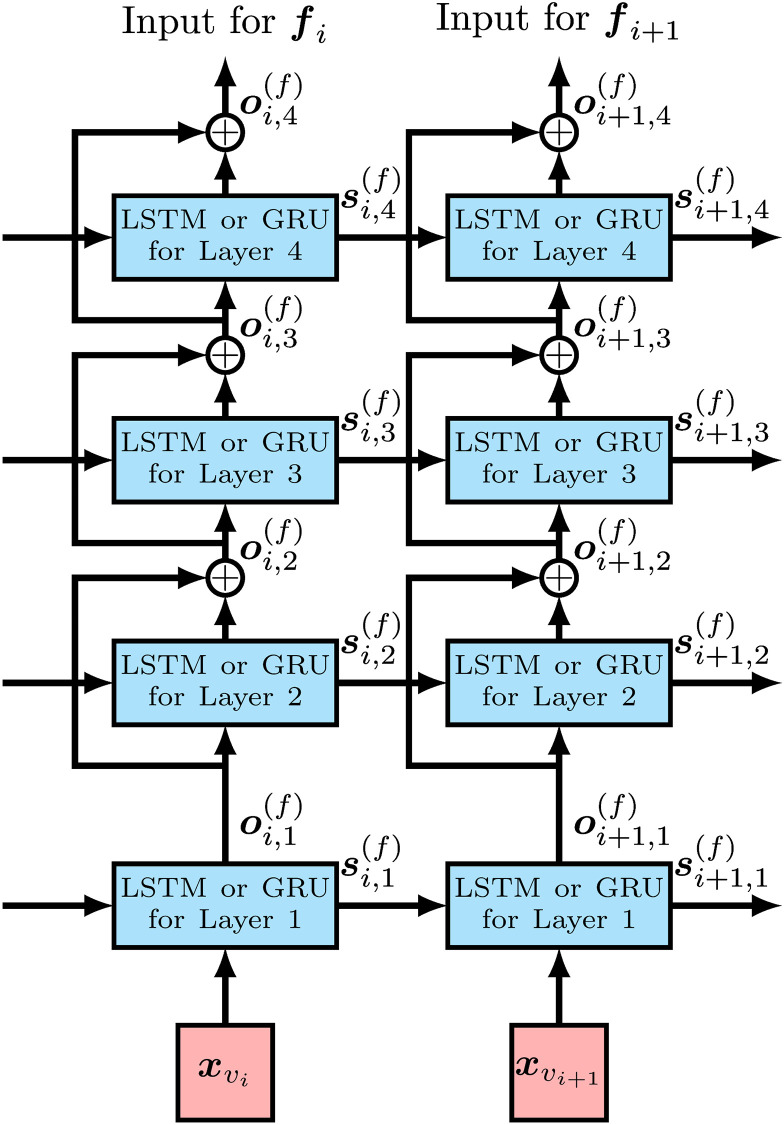
The structure of the forward RNN for each observed variant with residual connections for the case of four stacked RNN cells. $x_{v_{i}}$ and $x_{v_{i+1}}$ are input feature vectors for observed variants *v*_*i*_ and *v*_*i*+1_, respectively. $s_{i,j}^{\left(f\right)}$ is the state of the RNN cell of the *j*th layer for observed variant *v*_*i*_ and used as the input of the state for the RNN cell of the *j*th layer for observed variant *v*_*i*+1_. Circles with + represent the addition of tensors for residual connections. The output of the RNN cell of the top layer, ***o***_*i*,4_, is handled as the output of RNN for each observed variant.

Fig 4 shows the structure of the forward RNN for an observed variant with residual connections. Since the RNN model with residual connections is empirically not effective for the higher MAF model, we adopt residual connections only for the lower MAF model.

#### Self-attention

Attention was proposed to capture long range dependencies that are difficult to be captured by LSTM or GRU, in sequential data. Attention considers two types of elements, queries and keys, and generates a feature for each query using the keys. Attention can be considered among multiple sequences, and attention considered only in a sequence is called self-attention. In order to capture the long range dependencies, we consider self-attention for output vectors of our RNN model in a similar manner to a sentence embedding model proposed in [23]. While the sentence embedding model considers self-attention for concatenated output vectors of the forward and backward RNNs, we consider self-attention for output vectors of the forward and backward RNNs, independently, and additionally use the features from the self-attention for the forward and backward RNNs to estimate alleles for unobserved variants. We consider a simplified version of Transformer attention in [24, 25] as the model for the self-attention. Details of the proposed model with the self-attention are described in Section 1 of S1 Text in the supporting information.

### Input feature vectors for observed variants in a reference panel

Let *B* be a binary matrix representing a haplotype reference panel, where the *i*th row and *j*th column element indicates the allele of the *i*th haplotype in the *j*th variant. We first consider the *j*th column vector of *B* as a feature vector for an allele indicated by one at the *j*th variant. For observed variant *v*, we denote the feature vector for the allele indicated by one as $b_{v}^{1}$. We also let $b_{v}^{0}$ be the feature vector for the allele indicated by zero, in which the *i*th element takes one if the allele of the *i*th haplotype is indicated by zero, and zero otherwise. For example, let us consider the following allele pattern for a variant site with alleles ‘A’ and ‘T’ in the haplotype reference panel:

$$\begin{array}{c} {}[A,A,T,A,\ldots ,A,A,T,T]. \end{array}$$

If ‘A’ and ‘T’ are respectively indicated by one and zero, the corresponding binary representation is given by

$$\begin{array}{c} {}[1,1,0,1,\ldots ,1,1,0,0], \end{array}$$

and feature vectors $b_{v}^{1}$ and $b_{v}^{0}$ for allele ‘A’ and ‘T’ are given by [1, 1, 0, 1, …, 1, 1, 0, 0] and [0, 0, 1, 0, …, 0, 0, 1, 1], respectively. These feature vectors can be interpreted as a binary vector indicating which haplotype has the input allele for the variant. However, there exist two serious problems in the feature vectors; the feature vectors explicitly represent the haplotype reference panel, and the length of the feature vectors is too big as the input of the RNN since the number of the individuals in the haplotype reference panel is usually more than 1,000.

We thus adopt kernel principal component analysis (PCA) [26] as a dimensionality reduction technique for the feature vectors in order to resolve these two issues at the same time. Since the correlation of $b_{v}^{0}$ and $b_{v}^{1}$ is minus one, we apply kernel PCA only to the feature vectors for the alleles indicated by one: $b_{v_{1}}^{1},\ldots ,b_{v_{m}}^{1}$, in order to avoid the distortion in PCA results caused by the highly correlated variables. In order to obtain the dimensionally reduced feature vector of $b_{v}^{0}$, we project $b_{v}^{0}$ to the space from kernel PCA obtained for $b_{v}^{1}$. Given the original binary feature vector ***b***, the *i*th element of its dimensionally reduced feature vector is given by

$$\begin{array}{c} \frac{1}{\sqrt{d_{i}}}\sum_{j=1}^{m}u_{j}^{\left(i\right)}(k\left(b_{v_{j}}^{1},b\right)-\frac{1}{m}(k_{j}^{T}1+\sum_{k=1}^{m}k\left(b_{v_{k}}^{1},b\right))+\frac{1}{m^{2}}1^{T}K1), \end{array}$$

where *k*(⋅, ⋅) is a positive definite kernel, *K* is Gram matrix, ***k***_*i*_ is the *i*th column vector of *K*, *d*_*i*_ is the *i*th largest eigenvalue of the centered Gram matrix $\tilde{K}$, and $u_{j}^{\left(i\right)}$ is the *j*th element of the corresponding eigenvector of *d*_*i*_. The dimensionally reduced feature vector for variant *v* is used for ***x***_*v*_, the input feature vector for variant *v*. Details of the derivation for the above equation are in Section 2 of S1 Text in the supporting information.

### Training of the proposed model

We use Adam optimizer [27] to train the parameters of the proposed model. In order to avoid overfitting of the parameters, we consider averaged cross entropy losses and *R*^2^ values in the validation data as early stopping criteria. Note that *R*^2^ values are obtained by the squared correlation of true genotype counts and allele dosages as in [28]. In the practical trials, we find that the averaged *R*^2^ value in the validation data is suitable for lower MAF variants, while the cross entropy loss for the validation data is suitable for higher MAF variants. We thus use the cross entropy loss for the validation data as the early stopping criterion for training the higher MAF model, and the averaged *R*^2^ value in the validation data for the lower MAF model and the hybrid model in the following results. In the training step, we decrease the learning rate if the early stopping criterion is not updated in the specified number of iterations, which we call learning rate updating interval. Training stops if the learning rate gets less than the minimum learning rate or the iteration count reaches the maximum iteration count. Details of the training step are as follows:

1. Set iteration count *i* to 1 and set the best value for the early stopping criterion $\hat{c}$ to null.
2. Set learning rate *lr* and learning rate updating interval *li* to some initial values.
3. If *i* is larger than the maximum iteration count, finish training.
4. Update the model parameters by Adam optimizer with learning rate *lr* for randomly selected mini-batch data.
5. If *i* is divisible by validation interval *vi*, compute the following procedures:
  1. Calculate the current value for the early stopping criterion *c*.
  2. If $\hat{c}$ is null or *c* is better than $\hat{c}$, set $\hat{c}$ to *c*, save the current parameters, and set the last parameter saving step *i*_*s*_ to *i*.
  3. If *i* − *i*_*s*_ is larger than learning rate updating interval *li*:
    1. Divide learning rate *lr* by two.
    2. If learning rate *lr* is less than the minimum learning rate *lr*_min_, finish the training step.
    3. Divide learning rate updating interval *li* by two and round it down to an integer value.
    4. If learning rate updating interval *li* is less than the minimum learning rate updating interval *li*_min_, set *li* to *li*_min_.
    5. Set the last parameter saving step *i*_*s*_ to *i*.
    6. Restore the parameters to the previously saved parameters.
6. Increment *i* and go back to Step 2.

Since the local search in less space is expected for the smaller learning rate in the above procedures, we decrease the learning rate updating interval along with the learning rate. In our experiments, we set the initial learning rate to 0.0001, the minimum learning rate *lr*_min_ to 10^−7^, the initial learning rate updating interval to 5,000, the minimum learning rate updating interval *li*_min_ to 100, validation interval *vi* to 10, and the maximum iteration count to 100,000. We use randomly selected 500 haplotypes as the mini-batch data at each iteration.

**Fig 5:**
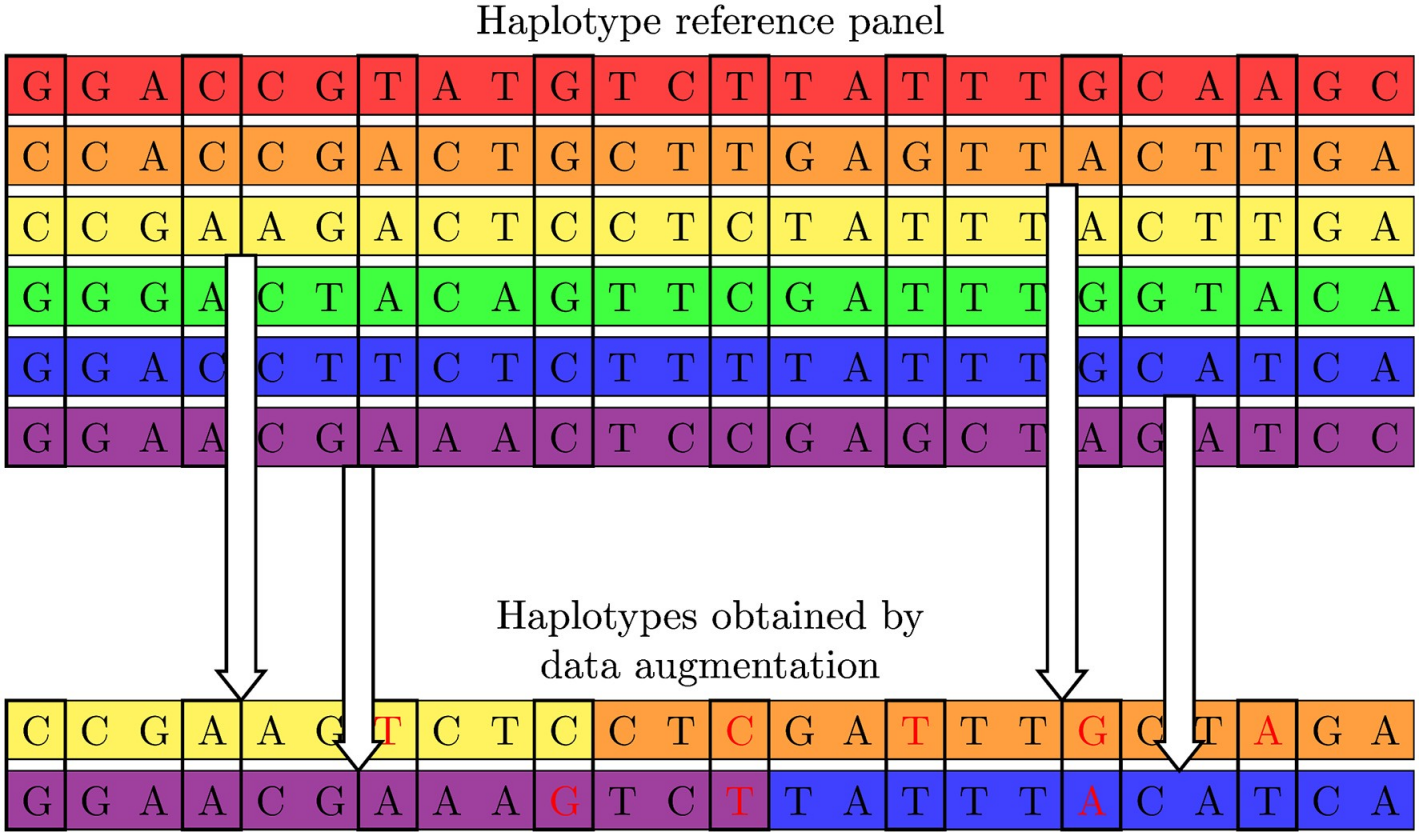
An illustration of the proposed data augmentation process, where new haplotypes are generated by applying mutations and recombinations for the haplotypes in the reference panel. Alleles surrounded by bold lines are those for the observed variants, and the mutations are applied only for the observed variants. Alleles in red are those mutated by the data augmentation process.

Existing imputation methods based on the Li and Stephens model are robust for haplotypes not in the haplotype reference panel because these methods consider mutations and recombinations for the reconstruction of the haplotypes not in the haplotype reference panel. In order to improve the robustness of the proposed method to haplotypes not in the haplotype reference panel, we propose a data augmentation process that generates new haplotypes by considering mutations and recombinations for the haplotypes in the reference panel for the training data. Fig 5 shows an example of the generation of new haplotypes by the proposed data augmentation process. The proposed data augmentation process applies mutations only for the observed variants according to the probability of mutations for a new haplotype in the Li and Stephens model. The probability of a mutation for each observed variant is given by 2*Neμ*/(4*Neμ* + *k*), where *Ne* is the effective population size and *μ* is the mutation rate. Similarly to the mutations, recombinations are applied according to the probability of recombinations for a new haplotype in the Li and Stephens model. The probability of a recombination between two variant sites is given by 1 − exp(−4*Neρd*_*j*_/*k*), where *ρ* is the recombination rate and *d*_*i*_ is the genetic distance between the *i*th and *i* + 1st variants. In our experiments, we set *Ne*, *μ* and *ρ* to 10,000, 10^−8^ and 10^−8^, respectively. Although *k* should be the number of the haplotypes in the reference panel, we set *k* to 25 to obtain the alleviated probabilities of the mutations and recombinations.

## Results and discussion

### Evaluation with 1KGP dataset

We use phased genotype data of 2,504 individuals for chromosome 22 from the phase 3 dataset of 1KGP [20]. We randomly select 100 individuals for test data and evaluated the imputation performance for the test data by using the phased genotype data of the remaining 2,404 individuals as the haplotype reference panel. In the test data, we extract genotype data for designed markers in SNP array and impute genotypes for the variants from the extracted genotype data by using the haplotype reference panel. We randomly select haplotypes for 100 individuals from the haplotype reference panel for validation data. We first examine the imputation accuracy of the proposed method for the following the number of layers *L*, the number of hidden units *H*, and RNN cell types:

- RNN cell type: LSTM or GRU
- The number of layers *L*: 2 or 4
- The number of hidden units *H*: 20 or 40

**Fig 6:**
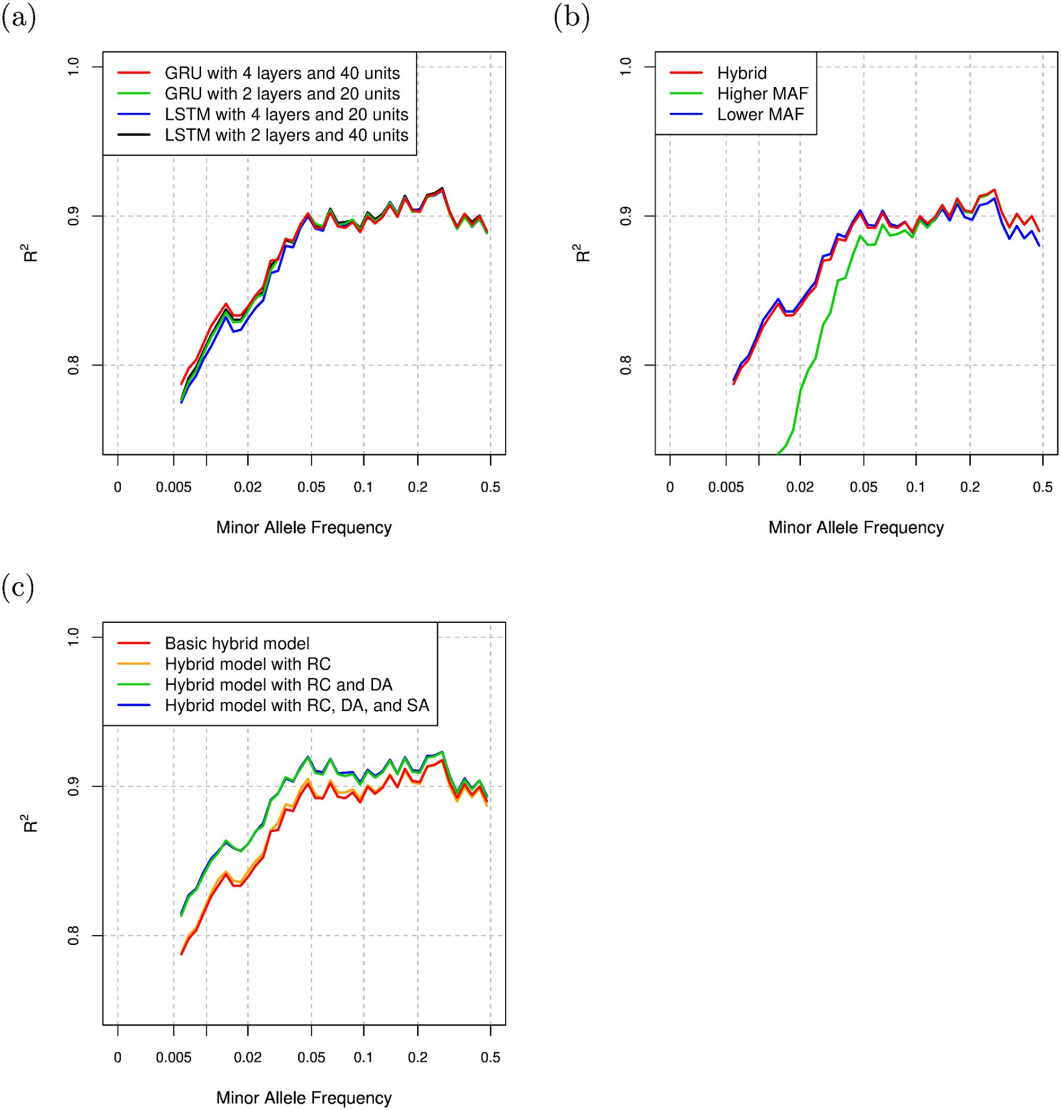
(a) Comparison of *R*^2^ values for the proposed method with several settings. (b) Comparison of *R*^2^ values for the proposed method with hybrid model, higher MAF model, and lower MAF model with the setting of GRU, 4 layers, and 40 hidden units. (c) Comparison of *R*^2^ values for the proposed method with and without residual connection (RC), data augmentation (DA), and self-attention (SA), where “Basic hybrid model” is the hybrid model without RC, DA, and SA.

The proposed method is implemented in Python 3, and TensorFlow (https://www.tensorflow.org/) is used for the implementation of RNN. Our implementation for the imputation with trained model parameters can be downloaded from a GitHub repository (https://github.com/kanamekojima/rnnimp). We extract genotypes for the designed markers in Infinium Omni2.5-8 BeadChip, which we hereafter call Omni2.5, in the test data. The number of the designed markers of Omni2.5 in chromosome 22 of the haplotype reference panel is 31,325, and 1,078,043 variants in the haplotype reference panel are not in the designed markers of Omni2.5 and used for the evaluation of imputation accuracy. It should be noted that we filter out the variants with MAF < 0.005 for imputation because the rare variants are not usually used for downstream analyses such as GWAS and high computation cost is required for imputing all the variant in the proposed method. As a positive definite kernel for feature extraction, we use the following homogeneous dot-product kernel [29]:

$$\begin{array}{c} k\left(b_{i},b_{j}\right)=∥b_{i}∥\;∥b_{j}∥\exp(\langle \frac{b_{i}}{\left||b_{i}|\right|},\frac{b_{j}}{\left||b_{j}|\right|}\rangle -1), \end{array}$$

where ||⋅|| indicates the L2 norm of the input vector and 〈⋅, ⋅〉 indicates the inner product of the two input vectors. For the loss function of the proposed method, *γ* is set to 0.75. We compare averaged *R*^2^ values in the validation data for the cases of using input feature vectors with the top 5, 10, and 20 principal component scores as shown in Table 1. In the comparison, the proposed model with GRU, 4 layers, and 40 hidden units is used, and the average *R*^2^ value for the case of the top 10 principal component scores is higher than that of the other cases although the length of input feature vectors *I* is not sensitive to the imputation accuracy. Based on the comparison, we use the top 10 principal component scores for the input feature vector in the following experiments. Table 2 shows the averaged *R*^2^ value in the validation data for each setting. Fig 6a shows the comparison of *R*^2^ values for the settings with minimum or maximum averaged *R*^2^ value for LSTM and GRU. The proposed model with GRU, 4 layers, and 40 hidden units gives the highest averaged *R*^2^ value in the validation data among the settings, and the comparison of averaged *R*^2^ values in the validation data is consistent with the results in the test data. In order to see the effectiveness of the hybrid model in the proposed method, we compare the *R*^2^ values in the results of hybrid model, higher MAF model, and lower MAF model. From the comparison of the *R*^2^ values in Fig 6b, the hybrid model is comparable with the higher MAF model in higher MAF range. In the low MAF range, the hybrid model is comparable with the lower MAF model and better than the model for higher MAF variants. Hence, the hybrid model is effective over the entire MAF range compared with the higher and lower MAF models. We also compare the proposed method with and without use of residual connections, self-attention, and the proposed data augmentation process in Fig 6c. The residual connections give a small improvement on the *R*^2^ values in the low MAF range, and the proposed data augmentation process gives a significant improvement on the *R*^2^ values in the entire MAF range. Although the self-attention also gives a small improvement on the *R*^2^ values, the proposed model with the self-attention requires approximately 10% more computational time as shown in Table A of S1 Text in the supporting information. Since the long-range dependencies captured by the self-attention seems to have a limited effect for imputation, we employ the proposed method without the self-attention in the following evaluations.

**Table 1:** Averaged *R*^2^ values in the validation data for the input feature vectors with size of 5, 10, and 20.

Size of Input Feature Vector	5	10	20
*R*^2^	0.8707	0.8708	0.8705

**Table 2:** Averaged *R*^2^ values in the validation data for several settings.

RNN cell	No. of Layers	No. of Hidden Units	*R*^2^
LSTM	2	20	0.8671
	2	40	0.8701
	4	20	0.8667
	4	40	0.8690
GRU	2	20	0.8673
	2	40	0.8702
	4	20	0.8674
	4	40	0.8708

**Fig 7:**
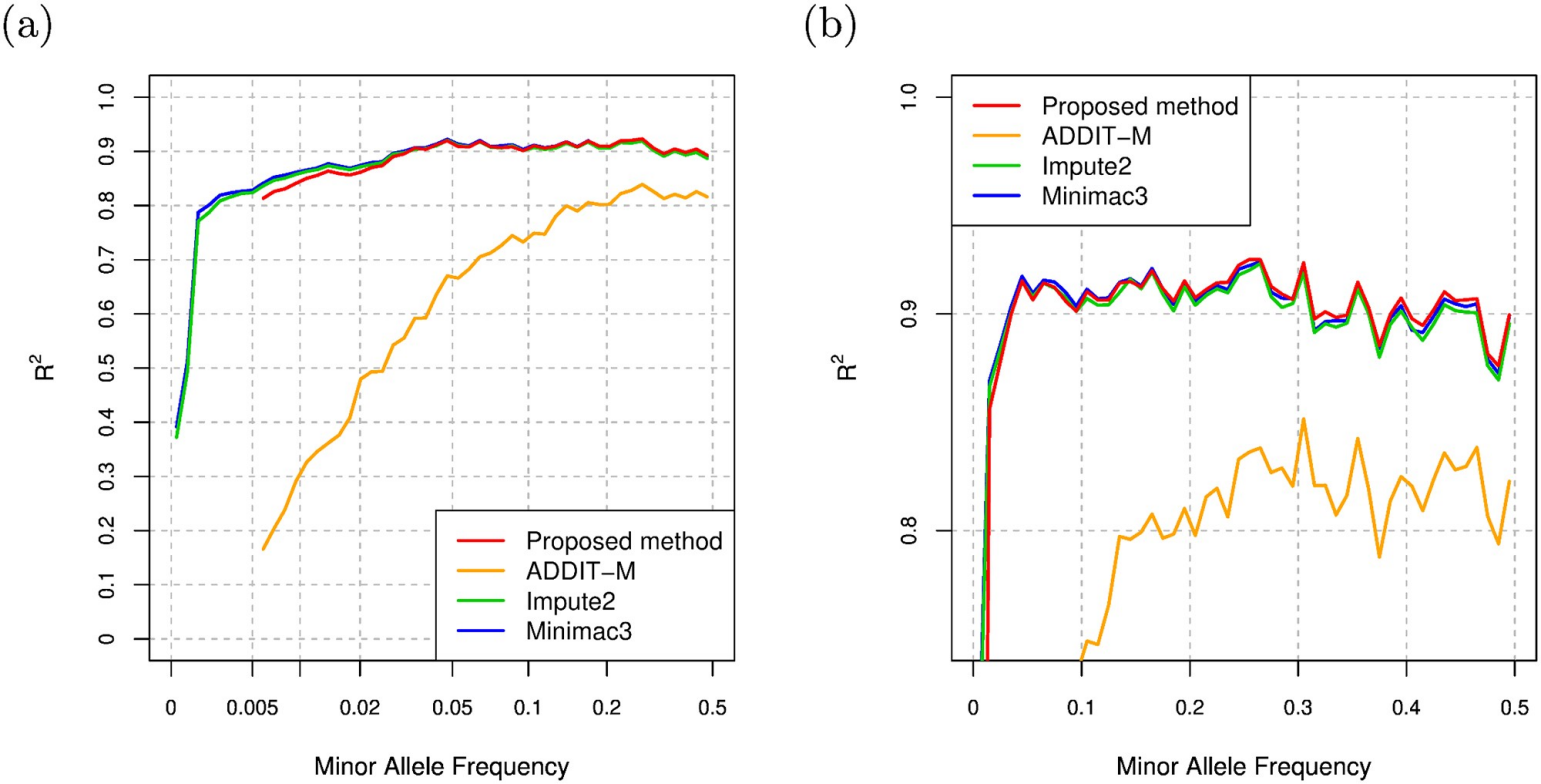
(a) Comparison of *R*^2^ values for the proposed method, ADDIT-M, Impute2, and Minimac3 for the 1KGP dataset. (b) Comparison of *R*^2^ values for the proposed method, ADDIT-M, Impute2, and Minimac3 in linear MAF scale and with zoom into higher *R*^2^ value for the 1KGP dataset.

We select Impute2 and Minimac3 as the representatives of existing imputation methods based on the Li and Stephens model, and compare the imputation performance of the proposed method and these methods. We set -k and k_hap options of Impute2 to the size of the haplotype reference panel to maximize the imputation accuracy. In addition to these two methods, we employ ADDIT-M as a supervised learning-based imputation method, in which genotype information is encoded to model parameters similarly to the proposed method. ADDIT-M estimates alleles of unobserved variants from alleles of observed variants using SVM trained with the haplotype reference panel. In the original Python implementation of ADDIT-M in the GitHub repository (https://github.com/NDBL/ADDIT), a regularization parameter, C, and a RBF kernel parameter, gamma, for SVM in scikit-learn, a Python machine learning library, are set to 0.001 and 0.8, respectively. Since ADDIT-M with SVM using the above hyperparameters always gives worse results than ADDIT-M with SVM using the default hyperparameters in scikit-learn in our experiments, we only consider ADDIT-M with SVM using the default hyperparameters in the following evaluation. The comparison of the imputation accuracy of ADDIT-M with the different SVM hyperparameters is summarized in Section 3 of S1 Text in the supporting information. For the proposed method, we use hybrid model with the setting of GRU, 4 layers, 40 hidden units, residual connections, and data augmentation. Fig 7a shows the comparison of the *R*^2^ values, and Fig 7b shows the comparison of the *R*^2^ values in linear MAF scale and with zoom into higher *R*^2^ value. We again note that *R*^2^ values are obtained by the squared correlation of true genotype counts and allele dosages as in [28]. Overall, the imputation accuracy of the proposed method is comparable with Impute2 and Minimac3 and better than that of ADDIT-M. Especially for the variants with MAF > 0.3, the proposed method is slightly better than Impute2. For the variants with MAF < 0.01, the *R*^2^ values of the proposed method are slightly lower than those of Impute2 and Minimac3 and better than those of ADDIT-M. While the Li and Stephens model considers a genealogy of haplotypes in the background in an approximate manner, the proposed method does not consider such a genetic background knowledge explicitly. The imputation accuracy of the proposed method thus tends to be lower than that of genotype imputation methods based on Li and Stephens model for low frequency variants such as the variants with MAF < 0.01.

We summarize the running time of the proposed method and the existing methods for imputation in Table 3. We measured the running time for imputation on Intel Xeon Silver 4116 CPU (2.10GHz) in a single process. In addition to imputation, the proposed method and ADDIT-M require the running time for training the model parameters. The proposed method, for instance, took a few days for training in a massively parallel computing system, and ADDIT-M took five hours in a single process, respectively. Since the trained model parameters can be used repeatedly for imputation on different datasets, we exclude the running time for training from the results in Table 3. Minimac3 also requires preprocessing for converting the haplotype reference panel in M3VCF format, which took one and a half hours in our experiment. Since the M3VCF format data also can be used repeatedly for imputation similarly to the model parameters for the proposed method and ADDIT-M, we exclude the running time for the preprocessing from the results in Table 3. Although the proposed method requires approximately two times as much running time as Impute2 for the imputation, the running time is still feasible for practical use. Since the running time of the proposed method is highly dependent on TensorFlow, the reduction of running time is expected along with the development of TensorFlow.

**Table 3:** Running time of the proposed method, ADDIT-M, Impute2, and Minimac3 for imputation using the 1KGP and HRC datasets.

Method	Running Time for 1KGP dataset	Running Time for HRC dataset
Proposed	25,119 [s]	19,438 [s]
ADDIT-M	842 [s]	1,566 [s]
Impute2	13,998 [s]	72,310 [s]
Minimac3	491 [s]	924 [s]

**Fig 8:**
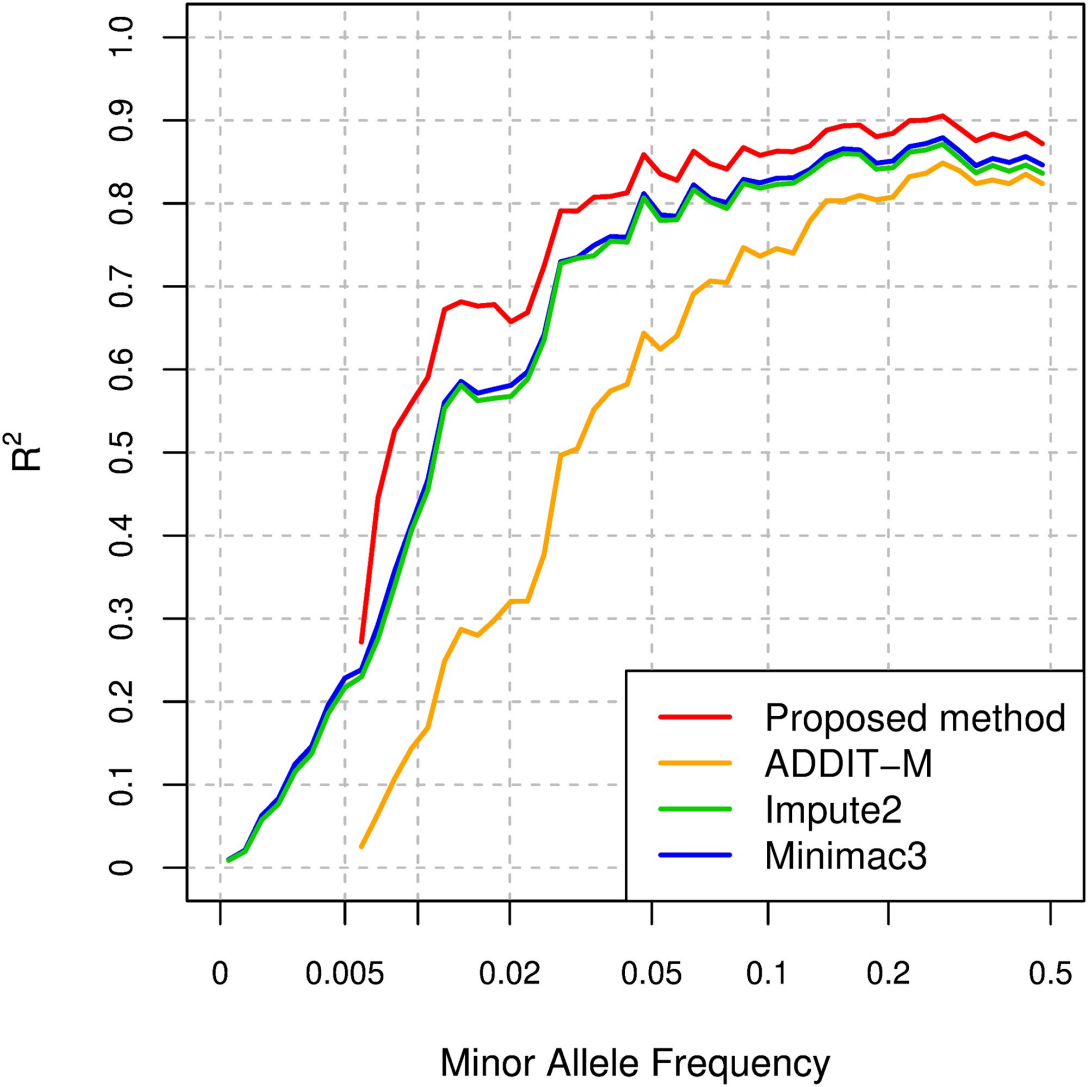
Comparison of *R*^2^ values for the proposed method, ADDIT-M, Impute2, and Minimac3 for EAS individuals.

We consider the case where the haplotypes of some individuals are not publicly available in an explicit form, but can be available in de-identified form; i.e., the haplotypes of the individuals cannot be used for Impute2 and Minimac3, but can be used for training the model parameters of the proposed method and ADDIT-M. In order to evaluate the imputation performance for the case, we randomly select 100 EAS individuals of 504 EAS individuals for the EAS test data, and prepare two types of haplotype reference panels: one is comprised of the remaining 2,404 individuals, and the other is comprised of 2,000 individuals and contains no EAS individuals. We use the former haplotype reference panel for training the proposed model and ADDIT-M, and use the latter haplotype reference panel for the imputation with Impute2 and Minimac3. In the evaluation, we consider Omni2.5 as the SNP array in the test data. For the proposed method, we also use hybrid model with the setting of GRU, 4 layers, 40 hidden units, residual connections, and data augmentation. Fig 8 shows the comparison of the *R*^2^ values for the test data in scaled MAF ranges. At least for the variants with MAF ≥ 0.005, the imputation accuracy of the proposed model is better than that of Impute2, Minimac3, and ADDIT-M in *R*^2^.

### Evaluation with HRC haplotype dataset

**Fig 9:**
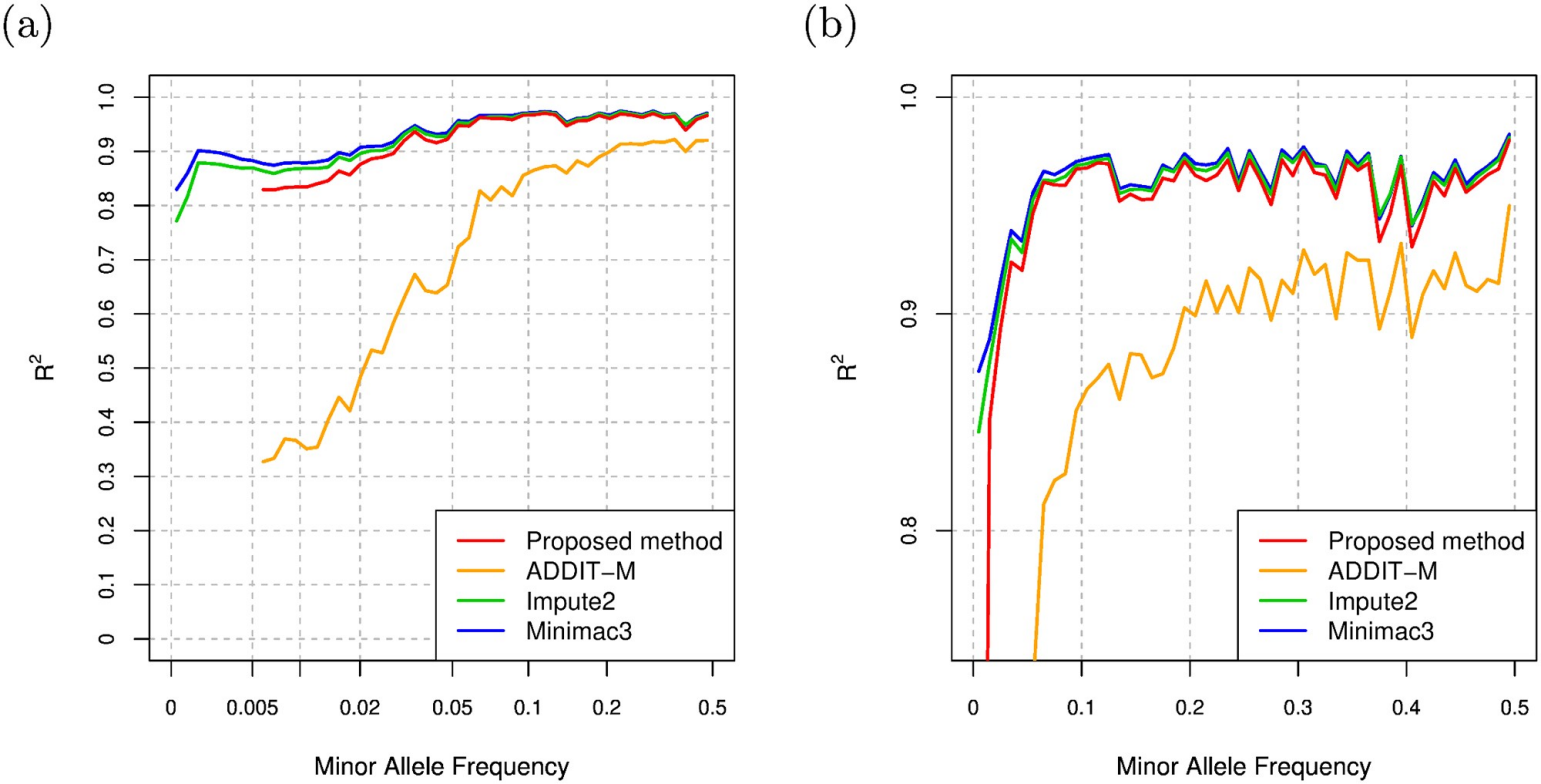
(a) Comparison of the *R*^2^ values of the proposed method, ADDIT-M, Impute2, and Minimac3 for the HRC dataset. (b) Comparison of the *R*^2^ values of the proposed method, ADDIT-M, Impute2, and Minimac3 in linear MAF scale and with zoom into higher *R*^2^ value for the HRC dataset.

HRC is a consortium for haplotypes from more than 20 cohort studies, and the number of the total haplotypes in HRC is currently 64,976. In addition to the collection of the haplotypes, HRC provides imputation servers in which imputation results based on its haplotype dataset can be obtained by uploading genotype datasets. In order to examine the performance of the proposed method for datasets larger than the dataset from 1KGP, we evaluate the proposed method using a haplotype dataset from HRC which is available at European Genome-phenome Archive (https://www.ebi.ac.uk/ega/studies/EGAS00001001710). The dataset is a subset of the HRC Release 1.1 and comprised of 54,330 haplotypes (27,165 individuals). Similarly to the evaluation with the 1KGP dataset, we randomly select 100 individuals for test data, and evaluate the imputation performance for the test data by using the phased genotype data of the remaining individuals as the haplotype reference panel. We randomly select haplotypes for 500 individuals from the haplotype reference panel for validation data. The number of the designed markers of Omni2.5 in chromosome22 of the haplotype reference panel is 31,441, and 493,103 variants in the haplotype reference panel are not in the designed markers of Omni2.5 and used for the evaluation of imputation accuracy. Fig 9a shows the comparison of the *R*^2^ values of the proposed method, Impute2, Minimac3, and ADDIT-M, and Fig 9b shows the comparison of the *R*^2^ values in linear MAF scale and with zoom into higher *R*^2^ value. Overall, the imputation accuracy of the proposed method is comparable with that of Impute2 and Minimac3. For variants with MAF < 0.01, *R*^2^ values of the proposed method is slightly lower than those of Impute2 and Minimac3 and higher than that of ADDIT-M similarly to the results for the 1KGP dataset.

We summarize the running time of the proposed method and the existing methods for imputation in Table 3. Similarly to the case of the 1KGP dataset, we measured the running time for imputation on Intel Xeon Silver 4116 CPU (2.10GHz) in a single process. For training the model parameters or preprocessing for the conversion to M3VCF format, the proposed method, ADDIT-M, and Minimac3 took a few days in a massively parallel computing system, six and a half days in a single process, and 5.8 hours in a single process, respectively. Since the trained model parameters and the M3VCF format data can be used repeatedly for imputation on different datasets, the running time for training and preprocessing is excluded from the results in Table 3. While the size of the haplotype reference panel influences the training time of the proposed method and ADDIT-M as well as the running time of Impute2 and Minimac3, the size does not influence the running time of the proposed method and ADDIT-M for imputation. The proposed method thus takes less running time than Impute2 for imputation in contrast to the 1KGP dataset although the running time of proposed method is still more than that of Minimac3. Since the HRC dataset contains less variant sites than the 1KGP dataset, the running time of the proposed method for the HRC dataset is less than that for the 1KGP dataset for imputation.

### Conclusion

In this study, we proposed a genotype imputation method for de-identified haplotype reference information by using the bidirectional RNN. Since the proposed method de-identifies the information of the haplotype reference panel by parameterizing it as its model parameters in the training step, the trained model parameters can be used publicly even when the original haplotype reference panel is not accessible publicly. In addition to the simple bidirectional RNN model, we considered the hybrid model, which is comprised of two types of models: one for higher MAF variants and the other for lower MAF variants. We also proposed a data augmentation process considering the mutations and recombinations, for more robust and accurate estimation.

Evaluation using the 1KGP dataset confirmed the effectiveness of the hybrid model by comparing it with the models for higher and lower variants. We also confirmed the effectiveness of the residual connections and the proposed data augmentation process.

While the proposed method handles the haplotype reference information in a de-identified form, we demonstrated that the proposed method could successfully provide accurate imputation results comparable with the results of existing imputation methods based on the Li and Stephens model, from the evaluation using the 1KGP and HRC datasets. We also compared the proposed method with an existing SVM-based imputation method, which can handle the haplotype reference panel in de-identified form as well, and confirmed the effectiveness of the proposed method as the imputation method for de-identified haplotype reference information. In order to show the reward of handling the de-identified haplotype reference information, we considered a scenario where some haplotypes were made available only in de-identified form for the haplotype reference panel. Under this scenario using the 1KGP dataset, the imputation accuracy of the proposed method was much higher than that of the existing methods in which some haplotype were not made available for the haplotype reference panel due to the accessibility limitation. Our RNN-based method is therefore considered to be quite promising to promote the data-sharing of sensitive genome data under the recent movement for the protection of individuals’ privacy.

## Supporting information

S1 Text Supporting information for “A genotype imputation method for de-identified haplotype reference information by using recurrent neural network”. (PDF)
