# Supplementary material for: A genotype imputation method for de-identified haplotype reference information by using recurrent neural network
Source: PLoS Comput Biol. 2020 Oct 1;16(10):e1008207. doi: 10.1371/journal.pcbi.1008207 (PMC7529210; doi:10.1371/journal.pcbi.1008207)
Supplement: S1 Text — (PDF) [file pcbi.1008207.s001.pdf]

Supporting information for "A genotype imputation method for de-identified haplotype reference information by using recurrent neural network"

Kaname Kojima[1,2], Shu Tadaka[1], Fumiki Katsuoka[1], Gen Tamiya[1,2], Masayuki Yamamoto[1,3,4], Kengo Kinoshita[1,4,5,6]

1 Tohoku Medical Megabank Organization, Tohoku University, Sendai, Miyagi, Japan
2 RIKEN Center for Advanced Intelligence Project, Chuo-ku, Tokyo, Japan
3 School of Medicine, Tohoku University, Sendai, Miyagi, Japan
4 Advanced Research Center for Innovations in Next-Generation Medicine, Tohoku University, Sendai, Miyagi, Japan
5 Graduate School of Information Sciences, Tohoku University, Sendai, Miyagi, Japan
6 Institute of Development, Aging and Cancer, Tohoku University, Sendai, Miyagi, Japan

1 Self-attention in proposed method

Attention was proposed to capture long range dependencies that are difficult to be captured by LSTM or GRU, in sequential data. Attention considers two types of elements, queries and keys, and generates a feature for each query using the keys. In general, attention can be considered among multiple sequences, and attention considered only in a sequence is called self-attention. In order to capture the long range dependencies, we consider self-attention for output vectors of our RNN model in a similar manner to a sentence embedding model proposed in [1]. While the sentence embedding model considers self-attention for concatenated output vectors of the forward and backward RNNs, we consider self-attention for output vectors of forward and backward RNNs, independently, and additionally use the features from the self-attention for the forward and backward RNNs to estimate alleles for unobserved variants. We first introduce self-attention for the forward RNN in the proposed model. Let $\boldsymbol{q}_{v_i}$ be a query associated with observed variant v_i. We also let $\boldsymbol{k}_{v_i}$ be a key associated with observed variant v_i. Both $\boldsymbol{q}_{v_i}$ and $\boldsymbol{k}_{v_i}$ are given by $\boldsymbol{o}_{i,L}^{(f)}$. We consider a simplified version of Transformer attention in [2] as the model of the self-attention. Let $\boldsymbol{o}_i^{A,(f)}$ be the output feature for query $\boldsymbol{q}_{v_i}$ given by the linear combination of the keys $\sum_j w_{ij}^A \boldsymbol{k}_{v_j}$, where w_{ij}^A is the attention weight for the query associated with v_i and key associated with v_j. Attention weight w_{ij}^A is proportional to the exponential value of the sum of the following four terms:

$$w_{ij}^A \propto \exp\left(\mathcal{E}_{ij}^1 + \mathcal{E}_{ij}^2 + \mathcal{E}_{ij}^3 + \mathcal{E}_{ij}^4\right),$$

where the sum of attention weights $\sum_j w_{ij}^A$ is constrained to 1. $\mathcal{E}_{ij}^1$ is a term representing the compatibility of $\boldsymbol{q}_{v_i}$ and $\boldsymbol{k}_{v_j}$ and given by $\boldsymbol{q}_{v_i}^T U^T V^C \boldsymbol{k}_{v_j}$, where U and V^C are learnable embedding matrices for the query and key, respectively. Both U and V^C are $E \times H$ matrices, where H is the length of $\boldsymbol{o}_{i,L}^{(f)}$ as in the main manuscript and E is set to 20 in our experiment. $\mathcal{E}_{ij}^2$ considers $\boldsymbol{q}_{v_i}$ and relative position of $\boldsymbol{q}_{v_i}$ and $\boldsymbol{k}_{v_j}$ and is given by $\boldsymbol{q}_{v_i}^T U^T V^R \boldsymbol{r}_{i-j}$, where $\boldsymbol{r}_{i-j}$ is a vector encoding the relative position

Table A. Running time of the proposed method with and without self-attention for imputation in the 1KGP dataset.

Method	Running Time
Proposed model with self-attention	28,332 [s]
Proposed model without self-attention	25,119 [s]

$i-j$ and V^R is a learnable embedding matrix for the encoded relative position. As in [3], we use sine and cosine functions of different wavelengths to represent the encoded relative position $\boldsymbol{r}_{i-j}$ as follows:

$$\boldsymbol{r}_{i-j} = \begin{bmatrix} \sin\left(2\pi(i-j)/10000^{1/n_r}\right) \\ \cos\left(2\pi(i-j)/10000^{1/n_r}\right) \\ \vdots \\ \sin\left(2\pi(i-j)/10000^{n_r/n_r}\right) \\ \cos\left(2\pi(i-j)/10000^{n_r/n_r}\right) \end{bmatrix},$$

where n_r is the half length of $\boldsymbol{r}_{i-j}$. Hence, V^R is a $E \times 2n_r$ matrix. n_r is set to 20 in our experiment. $\mathcal{E}^3_{ij}$ considers only $\boldsymbol{k}_{v_j}$ and is given by $\boldsymbol{u}^T V^C \boldsymbol{k}_{v_j}$, where $\boldsymbol{u}$ is a learnable vector of length E. $\mathcal{E}^4_{ij}$ also considers only the relative position of $\boldsymbol{q}_{v_i}$ and $\boldsymbol{k}_{v_j}$ and is given by $\boldsymbol{v}^T V^R \boldsymbol{r}_{i-j}$, where $\boldsymbol{v}$ is a learnable vector of E. The self-attention for the backward RNN is calculated by almost the same way as that for the forward RNN, and the different point in the calculation is the direction of the encoded relative position. Let $\boldsymbol{o}_i^{A,(b)}$ be the output feature for query $\boldsymbol{q}_{v_i}$ by the self-attention for the backward RNN. We additionally concatenate $\boldsymbol{o}_i^{A,(f)}$ and $\boldsymbol{o}_{i+1}^{A,(b)}$ to $\boldsymbol{f}_i$ to use them as the additional regressors of the softmax functions for the unobserved variants existing between observed variants v_i and v_{i+1}.

We apply the proposed method with and without the self-attention to impute randomly selected 100 individuals from the phase3 dataset of the 1000 Genome Project (1KGP) [4] using the remaining individuals of the dataset as a haplotype reference panel. The markers designed in Infinium Omni2.5-8 BeadChip are used as the observed variants, and the genotypes for the variants not in the designed markers are estimated from the observed variants for the randomly selected 100 individuals. Details of the dataset are in Results and Discussion Section of the main manuscript. Table A shows the running time of the proposed method with and without the self-attention for imputation in the 1KGP dataset. The use of the self-attention increases the running time for imputation by approximately 10% in the dataset. The comparison of the imputation accuracy is in Fig. 6c of the main manuscript.

2 Calculation of dimensionally reduced feature vectors

Let $\boldsymbol{b}_v^1$ be a binary feature vector of the allele indicated by one for variant v. Its ith element takes one if the allele of the ith haplotype in a haplotype reference panel is indicated by one and zero otherwise. We also let $\boldsymbol{b}_v^0$ be a binary feature vector of the allele indicated by zero in which the ith element takes one if the allele of the ith haplotype is indicated by zero and zero otherwise. We apply kernel principal component analysis (PCA) [5] to these feature vectors to obtain dimensionally reduced feature vectors. Since the correlation of $\boldsymbol{b}_v^0$ and $\boldsymbol{b}_v^1$ is minus one, we apply kernel PCA only to the feature vectors for the alleles indicated by one: $\boldsymbol{b}_{v_1}^1, \ldots, \boldsymbol{b}_{v_m}^1$, in order to

avoid the distortion in PCA results caused by the highly correlated variables. In order to obtain the dimensionally reduced feature vector of $\boldsymbol{b}_v^0$, we project $\boldsymbol{b}_v^0$ to the space from kernel PCA. We first describe the calculation of kernel PCA briefly, and then derive the projected values of $\boldsymbol{b}_v^0$ to the space from kernel PCA.

2.1 Calculation of kernel PCA

Let $k(\cdot,\cdot)$ and $\phi(\cdot)$ be a positive definite kernel and a map corresponding to k to reproducing kernel Hilbert space (RKHS) $\mathcal{H}$, respectively. From the property of RKHS, so called the kernel trick, the inner product $\left\langle \phi(\boldsymbol{b}_{v_i}^1), \phi(\boldsymbol{b}_{v_j}^1) \right\rangle$ is given by $k(\boldsymbol{b}_{v_i}^1, \boldsymbol{b}_{v_j}^1)$. The direction of the first principal component of kernel PCA for $\boldsymbol{b}_{v_1}^1, \ldots, \boldsymbol{b}_{v_m}^1$ is calculated as follows:

$$f^{(1)} = \arg\max_{f \in \mathcal{H}} \sum_{i=1}^{m} \left(\left\langle f, \tilde{\phi}(\boldsymbol{b}_{v_i}^1) \right\rangle \right)^2 \quad \text{s.t.} \quad ||f|| = 1,$$

where $\tilde{\phi}(\boldsymbol{b}_{v_i}^1) = \phi(\boldsymbol{b}_{v_i}^1) - \frac{1}{m}\sum_{j=1}^{m} \phi(\boldsymbol{b}_{v_j}^1)$ and $||\cdot||$ indicates the norm in RKHS $\mathcal{H}$. It is sufficient to consider the linear combination of $\tilde{\phi}(\boldsymbol{b}_{v_1}^1), \ldots, \tilde{\phi}(\boldsymbol{b}_{v_m}^1)$ for f since the directions orthogonal to all the $\tilde{\phi}(\boldsymbol{b}_{v_i}^1)$ do not contribute to the variance. Thus, the above formula can be rewritten as

$$\begin{aligned}
\boldsymbol{\alpha}^{(1)} &= \arg\max_{\boldsymbol{\alpha}} \sum_{i=1}^{m} \left(\left\langle \sum_{j=1}^{m} \alpha_j \tilde{\phi}(\boldsymbol{b}_{v_j}^1), \tilde{\phi}(\boldsymbol{b}_{v_i}^1) \right\rangle \right)^2 \\
&\qquad \text{s.t.} \quad \left|\left| \sum_{j=1}^{m} \alpha_j \tilde{\phi}(\boldsymbol{b}_{v_j}^1) \right|\right| = 1 \\
&= \arg\max_{\boldsymbol{\alpha}} \boldsymbol{\alpha}^T \tilde{K}^2 \boldsymbol{\alpha} \quad \text{s.t.} \quad \boldsymbol{\alpha}^T \tilde{K} \boldsymbol{\alpha} = 1,
\end{aligned}$$

where $\tilde{K}$ is a centered Gram matrix in which the ith row and jth column element is given by $\left\langle \tilde{\phi}(\boldsymbol{b}_{v_i}^1), \tilde{\phi}(\boldsymbol{b}_{v_j}^1) \right\rangle$. Let d_i and $\boldsymbol{u}^{(i)}$ be the ith largest eigenvalue of $\tilde{K}$ and its corresponding eigenvector, respectively. $\boldsymbol{\alpha}^{(1)}$ is given by $\frac{1}{\sqrt{d_1}}\boldsymbol{u}^{(1)}$, and hence $f^{(1)} = \frac{1}{\sqrt{d_1}} \sum_{i=1}^{m} u_i^{(1)} \tilde{\phi}\left(\boldsymbol{b}_{v_i}^1\right)$, where $u_i^{(1)}$ is the ith element of $\boldsymbol{u}^{(1)}$. The coefficients for the direction in the ith principal component $f^{(i)}$ is given by $\frac{1}{\sqrt{d_i}} \sum_{j=1}^{m} u_j^{(i)} \tilde{\phi}\left(\boldsymbol{b}_{v_j}^1\right)$ from a similar derivation to $f^{(1)}$.

2.2 Projection to kernel principal components

Let $\boldsymbol{b}$ be an original binary feature vector. The ith element of the dimensionally reduced feature vector for $\boldsymbol{b}$ is obtained by its projection to the ith kernel principal component, which is also called the ith kernel principal component score. The projected value of $\boldsymbol{b}$ to the ith kernel principal component is given as follows:

$$\begin{aligned}
&\left\langle f^{(i)}, \phi(\boldsymbol{b}) - \bar{\phi} \right\rangle \\
&= \left\langle \frac{1}{\sqrt{d_i}} \sum_{j=1}^{m} u_j^{(i)} \left(\phi(\boldsymbol{b}_{v_j}^1) - \bar{\phi} \right), \phi(\boldsymbol{b}) - \bar{\phi} \right\rangle \\
&= \frac{1}{\sqrt{d_i}} \sum_{j=1}^{m} u_j^{(i)} \left(k(\boldsymbol{b}_{v_j}^1, \boldsymbol{b}) - \frac{1}{m} \left(\boldsymbol{k}_j^T \mathbf{1} + \sum_{k=1}^{m} k(\boldsymbol{b}_{v_k}^1, \boldsymbol{b}) \right) + \frac{1}{m^2} \mathbf{1}^T K \mathbf{1} \right),
\end{aligned}$$

where $\bar{\phi} = \frac{1}{m}\sum_{i=1}^{m}\phi(\boldsymbol{b}_{v_i}^1)$, K is a Gram matrix in which the ith row and jth column element is $\left\langle \phi(\boldsymbol{b}_{v_i}^1), \phi(\boldsymbol{b}_{v_j}^1) \right\rangle$, and $\boldsymbol{k}_i$ is the ith column vector of K.

3 Comparison of ADDIT-M with two types of hyperparameters for Support Vector Machine

ADDIT-M is a supervised learning-based genotype imputation method which uses support vector machine (SVM) to estimate the alleles for unobserved variants from the alleles for observed variants [6]. In the original Python implementation of ADDIT-M in the GitHub repository (https://github.com/NDBL/ADDIT), a regularization parameter, C, and a RBF kernel parameter, gamma, for SVM in scikit-learn, a Python machine learning library, are set to 0.001 and 0.8, respectively. We here compare the imputation accuracy of ADDIT-M with two types of SVM hyperparameters: one is the hyperparameters in the original implementation of ADDIT-M and the other is the default SVM hyperparameters in scikit-learn. We consider the following three cases for comparing the imputation accuracy:

1. Imputation of randomly selected 100 individuals from the 1KGP dataset using the remaining individuals of the dataset as a haplotype reference panel.

2. Imputation of randomly selected 100 EAS individuals from the 1KGP dataset using the remaining individuals of the dataset as a haplotype reference panel.

3. Imputation of randomly selected 100 individuals from a haplotype dataset of the Haplotype Reference Consortium (HRC) [7] using the remaining individuals of the dataset as a haplotype reference panel.

The markers designed in Infinium Omni2.5-8 BeadChip are used as the observed variants, and the genotypes for the variants not in the designed markers are estimated from the observed variants for the randomly selected 100 individuals for each case. Details of the above datasets are in Results and Discussion Section of the main manuscript. Figures Aa, b, and c show the plots of the R^2 values for cases 1, 2, and 3 for ADDIT-M with the hyperparameters in the original implementation (Original) and ADDIT-M with the default hyperparameters (Default), respectively. The imputation accuracy of ADDIT-M with the default hyperparameters is better than that of ADDIT-M with the hyperparameters in the original implementation for all the cases.

References

1. Lin Z, Feng M, dos Santos CN, Yu M, Xiang B, Zhou B, Bengio Y. A structured self-attentive sentence embedding. The 5th International Conference on Learning Representations. 2017.

2. Zhu X, Cheng D, Zhang Z, Lin S, Dai J. An empirical study of spatial attention mechanisms in deep networks. Proceedings of the IEEE International Conference on Computer Vision. 2019;6688–6697.

3. Vaswani A, Shazeer N, Parmar N, Uszkoreit J, Jones L, Gomez AN, Kaiser Ł, Polosukhin I. Attention is all you need. Proceedings of the 31st International Conference on Neural Information Processing Systems. 2017;5998–6008.

4. 1000 Genomes Project Consortium *et al.* A global reference for human genetic variation. Nature. 2015;**526**(7571), 68–74.

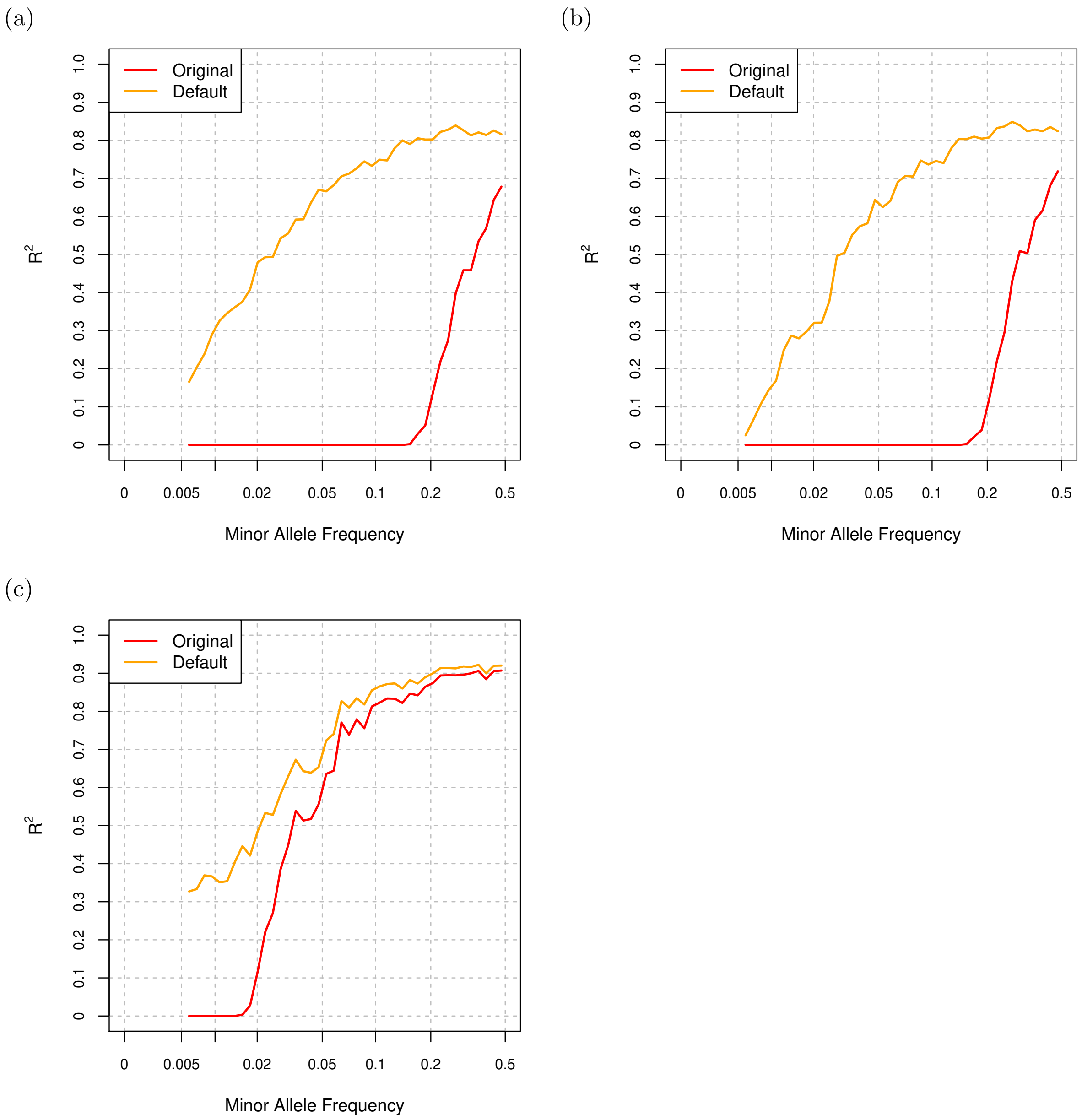

Figure A. (a) Comparison of R^2 values for randomly selected 100 individuals from the 1KGP dataset.(b) Comparison of R^2 values for randomly selected 100 EAS individuals from the 1KGP dataset.(c) Comparison of R^2 values for randomly selected 100 individuals from the HRC dataset.

5. Schölkopf, B, Smola A, Müller KR. Nonlinear component analysis as a kernel eigenvalue problem. Neural Computations. 1998;**5**(10), 1299-1319.

6. Choudhury O, Chakrabarty A, Emrich SJ. Highly accurate and efficient data-driven methods for genotype imputation. IEEE/ACM Transactions on Computational Biology and Bioinformatics. 2019;**16**(4), 1107–1116.

7. McCarthy S. *et al.* A reference panel of 64,976 haplotypes for genotype imputation. Nature Genetics. 2016;**48**(10), 1279–1283.
